# Association between blood lead levels and metabolic syndrome considering the effect of the thyroid-stimulating hormone based on the 2013 Korea National health and nutrition examination survey

**DOI:** 10.1371/journal.pone.0244821

**Published:** 2020-12-31

**Authors:** Ji Yoon Choi, Da-An Huh, Kyong Whan Moon

**Affiliations:** 1 Department of Health and Safety Convergence Science, Graduate School at Korea University, Seoul, Republic of Korea; 2 Environmental Health Research Division, National Institute of Environmental Research, Ministry of Environment, Incheon, Republic of Korea; 3 Department of Health Science, Graduate School at Korea University, Seoul, Republic of Korea; 4 BK21 FOUR R&E Center for Learning Health System & Department of Health and Environmental Science, Korea University, Seoul, Republic of Korea; Boston University School of Medicine, UNITED STATES

## Abstract

Imbalances in thyroid-stimulating hormone (TSH) levels are associated with metabolic syndrome (MetS), and the underlying mechanism is partly in alignment with that of lead exposure causing MetS. Many studies have reported the association between lead exposure and MetS, but no study has considered the possibility of TSH mediating lead's effect on MetS. Therefore, we aimed to examine the association between lead exposure and MetS considering TSH as a partial mediator. The data of 1,688 adults (age ≥19 years) from the Korea National Health and Nutrition Examination Survey in 2013 were analyzed. The prevalence of MetS in the Korean population was 21.9%, and the geometric mean of blood lead and serum TSH levels were 1.96 μg/dL and 2.17 μIU/mL, respectively. The associations between blood lead levels, serum TSH levels, and MetS were determined through a multiple logistic regression analysis. Blood lead levels were positively associated with high TSH levels (upper 25%) with an odds ratio (OR) and 95% confidence interval (CI) of 1.79 (1.24, 2.58) per doubled lead levels. The increase in blood lead and serum TSH levels both positively increased the odds of developing MetS. The OR of MetS per doubling of blood lead level was 1.53 (1.00, 2.35), and was not attenuated after adjusting for TSH levels. These findings suggest that higher levels of blood lead are positively associated with serum TSH levels and MetS. By exploring the role of TSH as a partial mediator between lead and MetS, we verified that lead exposure has an independent relationship with MetS, regardless of TSH levels.

## Introduction

Thyroid hormones play a key role in maintaining most of the basic metabolic processes in our body. They are significantly involved in the functions of the nervous, reproductive, and cardiovascular systems in both children and adults [1]. One of the most important regulators of thyroid function is the thyroid-stimulating hormone (TSH), which controls the serum levels of thyroid hormones by a negative feedback system [2]. Through numerous studies, a significant link has been established between TSH and metabolic syndrome (MetS), which is a cluster of abdominal obesity, hypertriglyceridemia, low high-density lipoprotein cholesterol (HDL-C) levels, high blood pressure, and fasting glucose disorder [3–5]. The association between TSH and MetS is partially explained by the participation of thyroid hormones in lipid metabolism. The normal biosynthesis of cholesterol is disturbed by thyroid disorders, leading to changes in serum lipid concentration [6]. In addition, atherogenic lipid alterations following thyroid dysfunction can also affect the vasculature leading to elevated blood pressure [7].

There are several environmental risk factors that can affect the homeostasis of TSH levels and induce some clinical disorders [8]. Lead is also one of the hazardous elements considered as a thyroid-disrupting chemical. A study suggested that lead exposure can induce functional impairment of the pituitary-thyroid axis and provoke the alteration of TSH levels [9]. This was supported through several epidemiologic studies which demonstrated the effect of lead exposure on TSH levels. The results from those studies presented significant associations between environmental exposure to lead and altered levels of TSH, even at low, general environmental concentrations [9–13].

Some studies have argued that environmental exposure to lead is associated with the occurrence of MetS [14, 15]. However, lead exposure can alter the levels of serum TSH, and an increase in TSH levels is significantly associated with the development of MetS. Moreover, lead and TSH share a common mechanism in the pathway causing MetS [16]. Exposure to lead disturbs systemic lipid metabolism and in turn induces adverse effects leading to MetS which overlaps with the effect of altered TSH levels contributing to the development of MetS [15, 16]. Thus, the impact of lead on MetS could be an indirect effect, considering the hypothetical causal chain where exposure to lead affects the TSH levels, and altered TSH levels thereby triggers the development of MetS. In other words, the influence of lead on the development of MetS could be partially or completely mediated by TSH. To date, no study has considered these three factors simultaneously, possibly overlooking the effect of TSH on the association between lead and MetS. Consequently, if TSH acts as an intermediate factor, previous studies might have overestimated the effect of lead exposure on MetS. Therefore, we hypothesized that altered levels of TSHs increase the risk of MetS and mediate the effect of lead on MetS.

**Fig 1:**
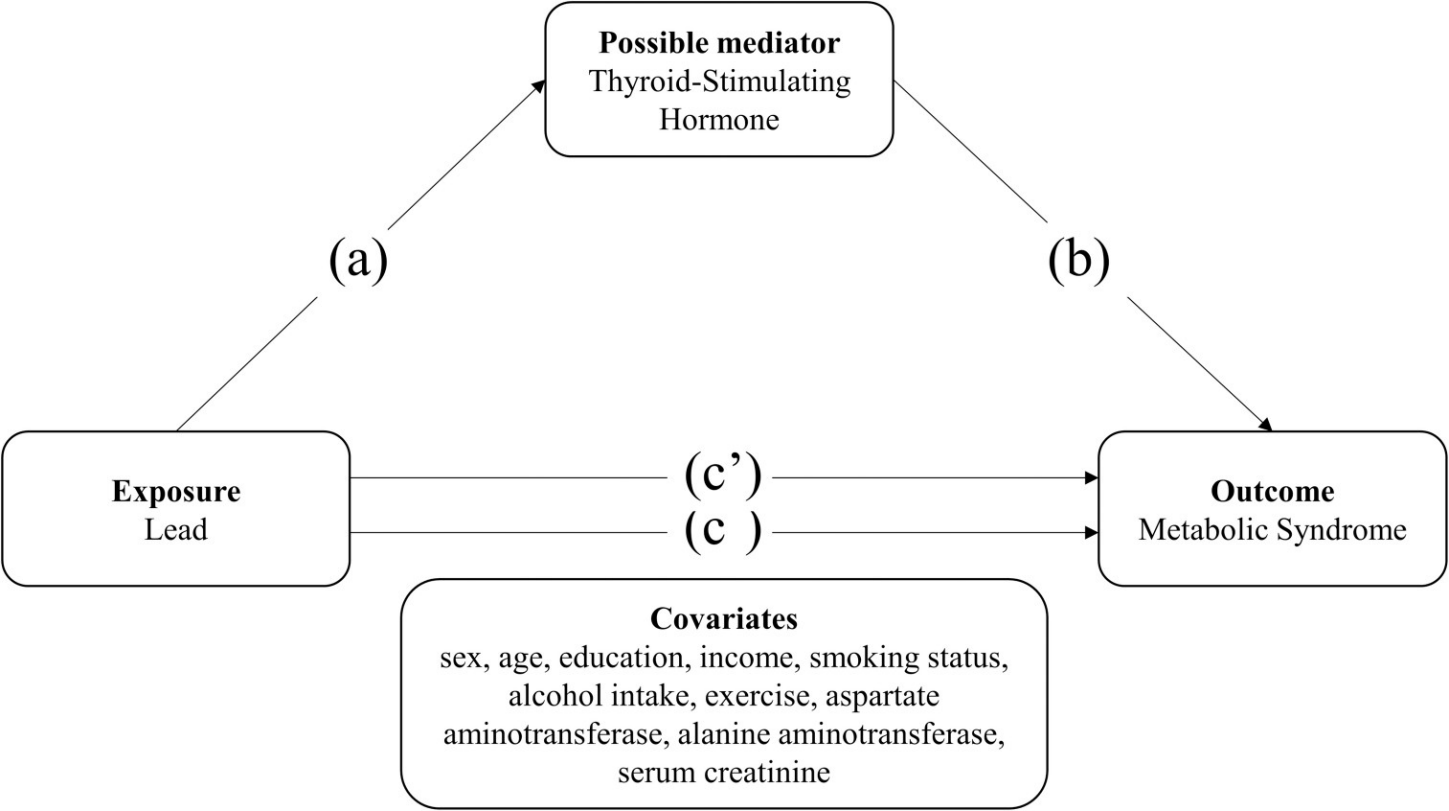
Diagram of the research model demonstrating the relationship between exposure and outcome variables: (a) association between blood lead and thyroid-stimulating hormone levels; (b) association between thyroid-stimulating hormone levels and metabolic syndrome; (c) association between blood lead levels and metabolic syndrome; (c’) association between blood lead levels and metabolic syndrome considering the effect of thyroid-stimulating hormone.

Therefore, this study aimed to determine the associations between environmental lead exposure, TSHs, and MetS in a large representative sample of the general Korean population. We also aimed to verify our hypothesis by examining the role of TSHs as a partial mediator in the association between lead and MetS (Fig 1).

## Methods

### Study population

The Korea National Health and Nutrition Examination Survey (KNHANES) is an ongoing series of cross-sectional surveys, which has been conducted by the Korea Center for Disease Control and Prevention since 1998. It is designed to obtain information about the health and nutrition status of the non-institutionalized Korean citizens from a representative sample of the Korean population. The survey is comprised of three parts: health and behavior interview, health examination, and nutrition survey. Health interviews and health examinations are conducted at the mobile examination center, and nutrition surveys are performed through household interviews.

**Fig 2:**
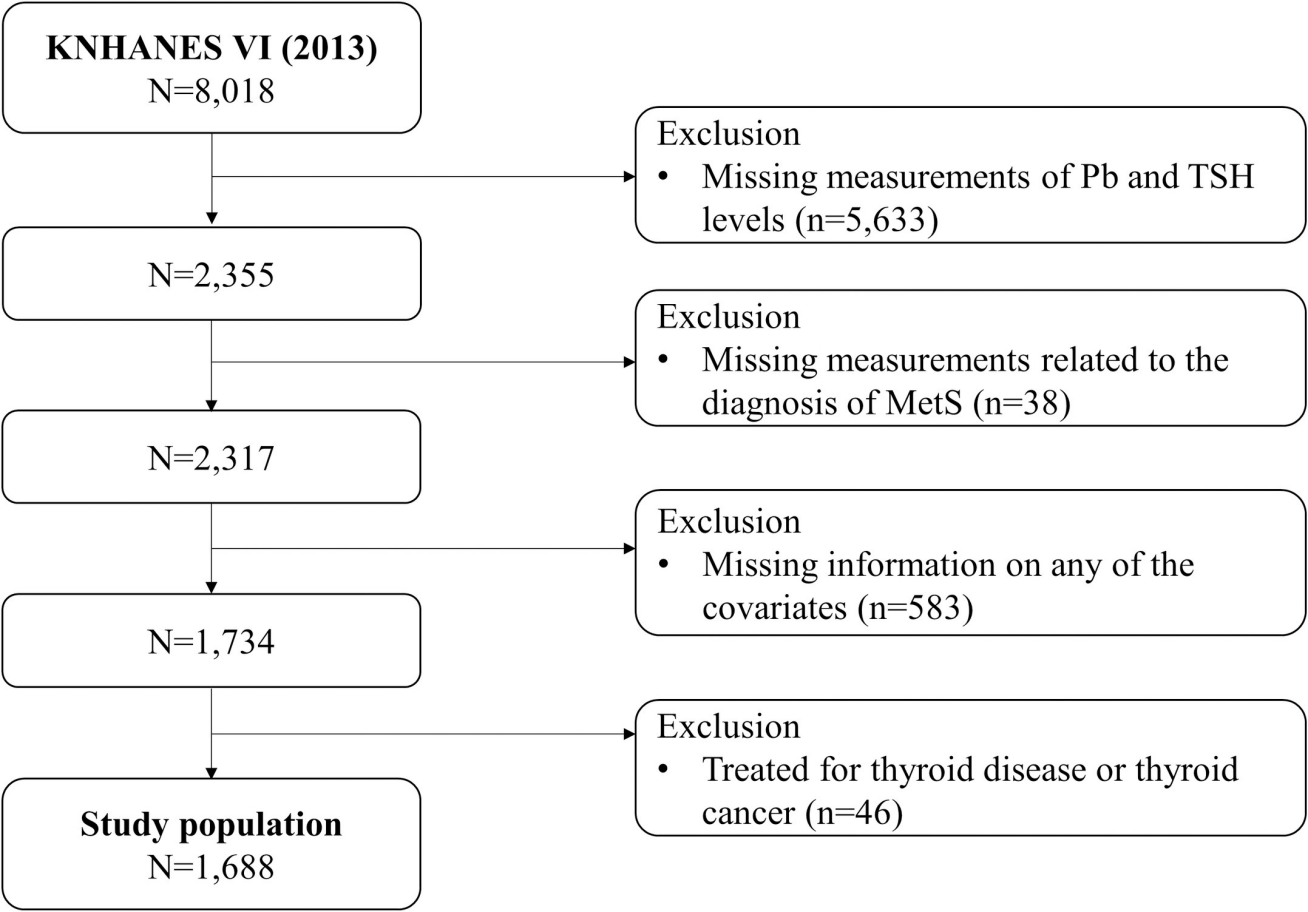
Exclusion criteria of study participants in the 2013 Korea national health and nutrition examination survey.

Among the KNHANES data sets, the present study used the data from the 2013 KNHANES. The 2013 survey was the only year that included both blood lead and serum TSH measurements in the health examination section, in addition to the measurement or survey data required for the diagnosis of MetS. Therefore, we used the data from the 2013 KNHANES to examine the association between lead exposure and MetS considering the effect of TSH levels. A total of 8,018 participants were included in the 2013 survey. Of those participants, 2,355 had measurement data on blood metal and TSH levels. We excluded individuals with missing information related to the diagnosis of MetS (n = 38), those with missing information on any other covariates (n = 583), and those who had been or are currently undertreatment for thyroid disease or thyroid cancer (n = 46). As a result, 1,688 participants aged 19 years or older were included in the final analysis (Fig 2).

### Blood lead levels

Heavy metal sampling was conducted in a subsample consisted of 2,400 individuals aged over 10 years, who were randomly selected from each survey unit according to sex and age. The blood lead levels were measured using graphite furnace atomic absorption spectrometry (model AAnalyst 600; PerkinElmer, Finland) at a central laboratory (NeoDin Medical Institute, Seoul, Korea). Internal quality assurance was achieved by ensuring quality control of all analytical equipment using the following standard reference materials: Whole Blood Metals Control (Bio-Rad, USA), Blood Metals Control (G-EQUAS, Germany), and pooled normal whole blood (self-manufactured). With regard to external quality control, the acceptable standards of precision and accuracy were met passing the German External Quality Assessment Scheme, CDC Lead and Multielement Proficiency Program, and Korea Occupational Safety and Health Agency’s Quality Control of Special Health Checks. The limit of detection for lead was 0.223 μg/dL according to the final report of the KNHANES 2013 clinical examinations.

### Thyroid-stimulating hormone levels

The levels of serum TSH in a subsample of 2,400 individuals aged over 10 years were analyzed. Approximately 15 mL of blood was collected, and within 30 minutes the serum was separated and then transferred to the testing facility. Serum TSH levels were measured with an electrochemiluminescence immunoassay (Cobas8000 E-602; Roche, Germany) at the central laboratory (NeoDin Medical Institute, Seoul, Korea) within 24 hours after sampling. An E-TSH kit (Roche Diagnostics) was used for measuring the levels of TSH, and the reference range was 0.35–5.50 mIU/L. The results reported met specifications for accuracy, general chemistry, special immunology, and ligand established by the quality control and assurance program of the College of American Pathologists.

### Metabolic syndrome

MetS was diagnosed according to the criteria of the revised National Cholesterol Education Programme Adult Treatment Panel Ⅲ (NCEP ATP Ⅲ) proposed in 2005 by the American Heart Association and National Heart, Lung and Blood Institute, with a modified standard for defining abdominal obesity based on the criteria suggested by the Korean Society of Obesity [17, 18]. Based on the criteria of the revised NCEP ATP Ⅲ, MetS was defined as the presence of at least three of the following features: 1) abdominal obesity (waist circumference: ≥90 cm in men and ≥85 cm in women, 2) elevated triglycerides (serum triglyceride (TG) levels: >150 mg/dL) or receiving treatment for elevated triglycerides, 3) HDL-C levels less than 40 mg/dL in men and 50 mg/dL in women or receiving treatment for reduced HDL-C levels, 4) elevated blood pressure (systolic blood pressure: >130 mmHg or diastolic blood pressure: >85 mmHg) or receiving antihypertensive treatment, and 5) fasting glucose disorder (fasting glucose: >100 mg/dL) or receiving treatment after being diagnosed with diabetes.

### Covariates

The potential confounders that had an effect on the association between lead exposure and MetS were sex, age, education levels, household income, exercise, smoking status, alcohol intake, aspartate aminotransferase (AST) levels, alanine aminotransferase (ALT) levels, serum creatinine levels, and blood mercury and cadmium levels. The level of education was classified into three categories according to the individual’s highest level of education: less than high school graduation, high school graduate, and college or more. Household income was divided by quartiles. People who responded that they do not perform moderate levels of exercise more than 5 days a week, 30 minutes per day, were classified into the little exercise group, while those who responded that they perform moderate exercise more than 5 days a week, 30 minutes per day, were classified into the moderate exercise group. People who responded that they perform strenuous exercise more than 3 days a week, 20 minutes per day, as well as those who answered positively to both questions were classified into the vigorous exercise group [19, 20]. Smoking status and alcohol intake were divided into three groups: never, former, and current smoker or drinker, respectively [19]. In addition to the variables previously mentioned, creatinine-adjusted urinary iodine was also included as a covariate when the association with TSH was being considered.

### Statistical analysis

In this study, we performed statistical analyses to determine the association between lead, TSH, and MetS and furthermore tested for mediation following the methods of Navas-Acien [21] and Agarwal [22]. Referring to their methods, the four step approach proposed by Baron and Kenny was used [23]. In our case, TSH was hypothesized to be the intervening variable causing a mediation effect on the association between lead and MetS. Thus, we first examined the association among the following variables: (a) blood lead and serum TSH levels, (b) serum TSH levels and MetS, and (c) blood lead levels and MetS (Fig 1). Moreover, to verify the possible mediating effect of serum TSH levels, the association between blood lead levels and MetS was examined after further adjustment for serum TSH levels (c’).

The associations between the three variables were determined by obtaining the odds ratios (ORs) and their 95% confidence intervals (CI) using multiple logistic regression analysis. First, we examined the association between blood lead levels and high TSH levels, which was defined as the upper 25% of the sample’s TSH level distribution. Blood lead levels were log-transformed before analysis due to their skewed distribution and examined as continuous variables. The participants were also subdivided into quintiles based on their blood lead levels; these variables were included in the logistic regression models as categorical variables. The model was adjusted for sex, age, education, income, smoking status, creatinine-adjusted urinary iodine level, body mass index (BMI), mercury level, and cadmium level. Second, the association between serum TSH levels and MetS was determined. Along with the prevalence of MetS, the correlations between the diagnostic components of MetS and serum TSH levels were determined. Serum TSH levels were log-transformed prior to analysis and categorized by quintiles. All models were adjusted for sex, age, education, income, smoking status, alcohol intake, exercise, AST level, ALT level, serum creatinine level, and creatinine-adjusted urinary iodine level. Finally, the association between blood lead and MetS was examined. The models were progressively adjusted for covariates and then further adjusted for serum TSH and creatinine-adjusted urinary creatinine to test for mediation effects.

### Ethics statement

The Korea National Health and Nutrition Examination Survey (KNHANES) was approved by the institutional review board of Korea Centers for Disease Control and Prevention (KCDC), and the approval codes from 2013 are as follows: 2013-07CON-03-4C and 2013-12EXP-03-5C. We used secondary data provided from the KNHANES for this study.

## Results

Table 1 demonstrates the distribution of the basic characteristics of 1,688 study participants according to MetS status. The weighted prevalence of MetS in the Korean population was 21.9%. People with MetS were significantly older and had higher BMI levels. The proportion of people with lower education levels were higher in the MetS group, and people in the non-MetS group had higher household incomes. The MetS group showed higher proportions of individuals with little and moderate exercise levels. Blood levels of heavy metals and TSH were significantly higher in individuals with MetS.

**Table 1:** Demographic and clinical characteristics of the study participants according to metabolic syndrome status (unweighted).

Characteristic	No metabolic syndrome (n = 1,339)	Metabolic syndrome (n = 349)	p value
Age (years)	42.2 (0.40)	54.1 (0.64)	<0.001
Sex (male n, %)	693 (51.8)	192 (55.0)	0.479
Body mass index (kg/m^2^)	23.1 (0.11)	26.5 (0.22)	<0.001
Education (n, %)			<0.001
<High school	246 (18.4)	141 (40.4)	
High school graduate	394 (29.4)	112 (32.1)	
≥College	699 (52.2)	96 (27.5)	
Income, 10,000 \ (n, %)			<0.001
1^st^ quartile (<75.00)	159 (11.9)	88 (25.2)	
2^nd^ quartile (75.00–150.00)	367 (27.4)	90 (25.8)	
3^rd^ quartile (150.00–246.31)	361 (27.0)	99 (28.4)	
4^th^ quartile (≥246.31)	452 (33.8)	72 (20.6)	
Exercise (n, %)			0.027
Little	1,033 (77.1)	287 (82.2)	
Moderate	33 (2.5)	9 (2.6)	
Vigorous	273 (20.4)	53 (15.2)	
Smoking status (n, %)			0.304
Never	750 (56.0)	183 (52.4)	
Former smoker	253 (18.9)	74 (21.2)	
Current smoker	336 (25.1)	92 (26.4)	
Alcohol intake (n, %)			0.006
Never	97 (7.2)	40 (11.5)	
Former drinker	173 (12.9)	60 (17.2)	
Current drinker	1,069 (79.8)	249 (71.3)	
AST (IU/L)	19.7 (1.01)	22.9 (1.02)	<0.001
ALT (IU/L)	17.5 (1.02)	23.8 (1.03)	<0.001
Serum creatinine (mg/dL)	0.83 (1.01)	0.85 (1.01)	0.049
Blood lead (μg/dL)	1.89 (1.02)	2.23 (1.02)	<0.001
Blood mercury (μg/L)	3.20 (1.02)	3.80 (1.04)	<0.001
Blood cadmium (μg/L)	0.81 (1.02)	1.00 (1.03)	<0.001
Serum TSH (uIU/mL)	2.13 (1.02)	2.32 (1.04)	0.034
Creatinine-adjusted urinary iodine (μg/g creatinine)	2.40 (1.04)	2.44 (1.07)	0.824

Note: AST, aspartate aminotransferase; ALT, alanine aminotransferase; TSH, thyroid–stimulating hormone.

Data are presented as arithmetic mean (SE), geometric mean (SE), or n (%).

**Fig 3:**
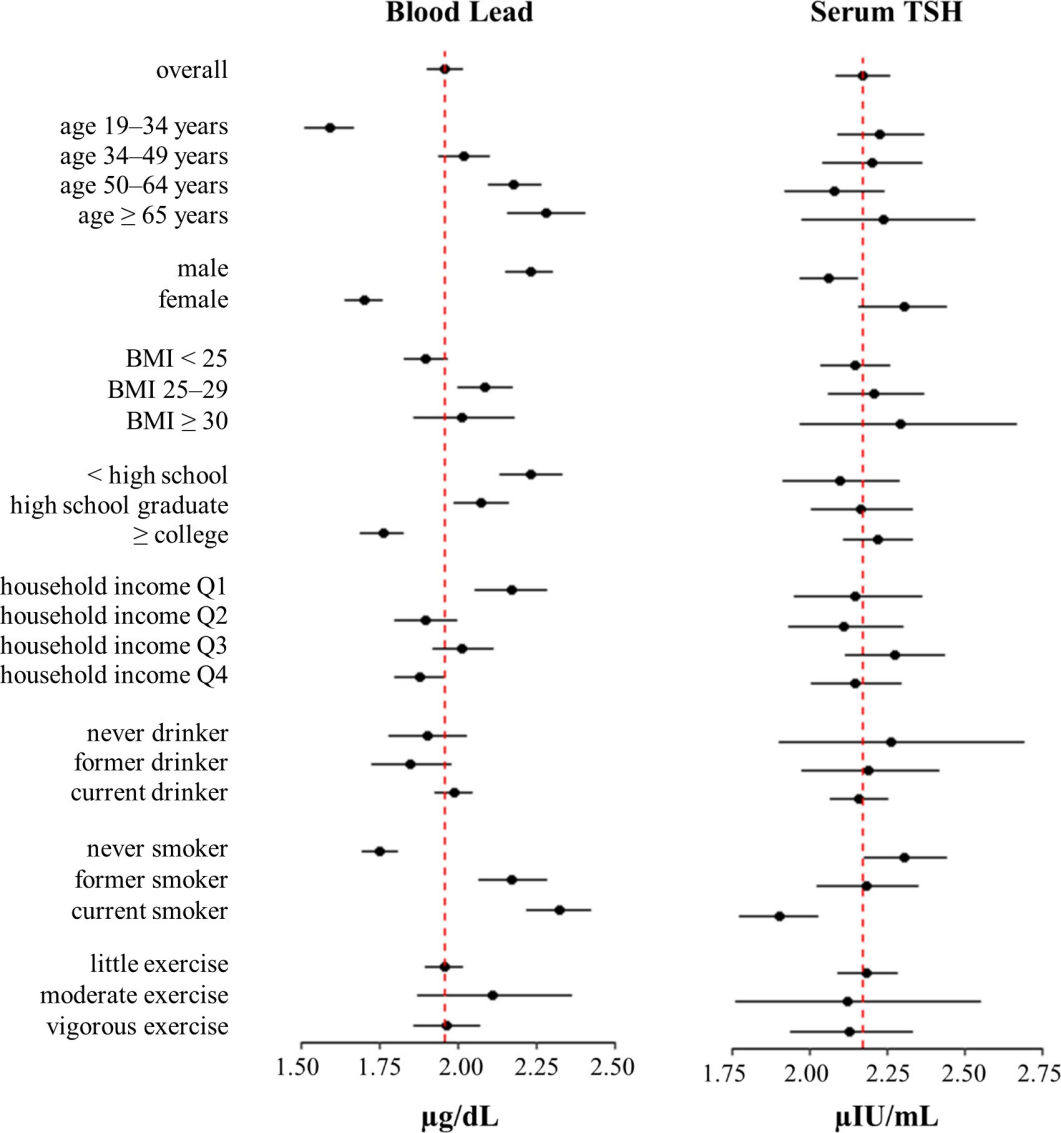
Geometric mean of blood lead and thyroid-stimulating hormone levels (95% confidence interval) according to participant characteristics. Points represent geometric means, horizontal lines represent 95% confidence intervals, and the dotted vertical line represents the geometric mean in the overall study sample.

The distribution of blood lead and serum TSH levels according to the characteristics of the study participants are shown in Fig 3. The points indicate the geometric means, while the horizontal lines display the 95% confidence intervals of the geometric means. The weighted geometric means of blood lead and serum TSH levels were 1.96 μg/dL and 2.17 μIU/mL, respectively, and are represented by the dotted vertical line. Blood lead levels increased by age, were higher in men than in women, and increased by smoking status. Serum TSH levels were higher in women than in men and increased with higher BMI levels.

The ORs for high TSH levels (upper 25%) based on log-transformed lead levels and lead quintiles are shown in Table 2. Lead levels were positively associated with high TSH levels after adjusting for the potential confounders. The OR (95% CI) for the occurrence of high TSH levels per one unit increase of log-transformed lead levels was 1.79 (1.24, 2.58). The ORs for high TSH levels also increased according to the lead quintiles compared to the lowest quintile, and there was a significant increase in the trend for the OR (p for trend = 0.028).

**Table 2:** ORs (95% CIs) of high TSH (upper 25%) according to log-transformed lead levels and lead quintiles.

	n	Model 1^a^	Model 2^b^
Lead (μg/dL)			
Per doubling of lead level		1.04 (0.80, 1.36)	1.79 (1.24, 2.58)
Lead quintiles (μg/dL)			
Q1 (<1.400)	337	Reference	Reference
Q2 (1.400–1.768)	338	0.94 (0.64, 1.37)	1.09 (0.73, 1.62)
Q3 (1.769–2.194)	338	0.90 (0.59, 1.35)	1.24 (0.79, 1.96)
Q4 (2.196–2.737)	338	0.93 (0.63, 1.37)	1.34 (0.85, 2.10)
Q5 (≥2.741)	337	0.92 (0.64, 1.31)	1.64 (1.04, 2.61)
p for trend		0.649	0.028

Note: OR, odds ratio; CI, confidence interval; TSH, thyroid–stimulating hormone.

^a^Model 1 was unadjusted for any covariate.

^b^Model 2 was adjusted for sex, age, education, income, smoking status, creatinine-adjusted urinary iodine level, BMI, mercury level, and cadmium level.

Table 3 demonstrates the association between MetS risk and serum TSH levels. The prevalence of MetS increased with higher TSH levels. The OR (95% CIs) for MetS one unit of increase in ln-TSH levels was 1.25 (1.03, 1.51). The ORs for possessing the clinical aspects related to each of the five diagnostic components of MetS were also evaluated according to the log-transformed serum TSH levels. Although the associations with TSH levels were not significant for all components, the ORs for having clinical disorders related to the diagnostic components of MetS indicated positive associations between the diagnostic components and TSH levels.

**Table 3:** ORs (95% CIs) of the diagnostic components of metabolic syndrome according to log-transformed serum TSH levels and TSH quintiles.

	n	Abdominal obesity	High TG	Low HDL-C	High BP	Fasting glucose disorder	MetS
TSH (uIU/mL)							
Per doubling of TSH levels		1.24 (1.02, 1.51)	1.15 (0.97, 1.35)	0.97 (0.84, 1.13)	1.04 (0.86, 1.26)	1.01 (0.86, 1.19)	1.25 (1.03, 1.51)
TSH quintiles (uIU/mL)							
Q1 (<1.36)	334	Reference	Reference	Reference	Reference	Reference	Reference
Q2 (1.36–1.92)	335	0.84 (0.54, 1.29)	1.08 (0.75, 1.56)	0.68 (0.43, 1.05)	1.27 (0.79, 2.04)	1.04 (0.68, 1.59)	1.05 (0.64, 1.74)
Q3 (1.92–2.59)	343	1.04 (0.66, 1.65)	0.96 (0.62, 1.49)	1.14 (0.74, 1.74)	1.03 (0.63, 1.70)	0.97 (0.61, 1.54)	1.32 (0.77, 2.25)
Q4 (2.59–3.63)	337	0.87 (0.56, 1.37)	1.27 (0.90, 1.81)	0.88 (0.58, 1.33)	1.28 (0.80, 2.05)	1.11 (0.74, 1.66)	1.38 (0.87, 2.19)
Q5 (≥3.63)	339	1.19 (0.75, 1.89)	1.07 (0.72, 1.60)	1.06 (0.72, 1.55)	1.15 (0.74, 1.80)	0.94 (0.62, 1.40)	1.19 (0.78, 1.80)
p for trend		0.462	0.521	0.420	0.592	0.883	0.178

Note: OR, odds ratio; CI, confidence interval; TSH, thyroid–stimulating hormone; TG, triglyceride; HDL-C, high-density lipoprotein cholesterol; BP, blood pressure; MetS, metabolic syndrome.

All models were adjusted for sex, age, education, income, smoking status, alcohol intake, exercise, aspartate aminotransferase level, alanine aminotransferase level, serum creatinine level, and creatinine-adjusted urinary iodine level.

The association between MetS risk and blood lead concentration is presented in Table 4. The prevalence of MetS increased with higher lead levels with an OR (95% CI) of 2.66 (1.90, 3.71). After adjusting for other confounders, the OR decreased but remained significant. The adjusted OR (95% CI) of MetS per one unit increase of log- transformed lead level was 1.53 (1.00, 2.35). Further adjustment for serum TSH levels did not significantly attenuate the OR (1.52 [0.98, 2.34]), and the OR remained almost constant at 1.53 (0.99, 2.37) after additional adjustment for creatinine-adjusted urinary iodine.

**Table 4:** ORs (95% CIs) of MetS according to log-transformed lead levels and lead quintiles.

	n	Model 1^a^	Model 2^b^	Model 3^c^	Model 4^d^
Lead (μg/dL)					
Per doubling of lead levels		2.66 (1.90, 3.71)	1.53 (1.00, 2.35)	1.52 (0.98, 2.34)	1.53 (0.99, 2.37)
Lead quintiles (μg/dL)					
Q1 (<1.400)	337	Reference	Reference	Reference	Reference
Q2 (1.400–1.768)	338	1.58 (0.95, 2.63)	1.26 (0.71, 2.24)	1.26 (0.71, 2.24)	1.27 (0.71, 2.27)
Q3 (1.769–2.194)	338	2.74 (1.69, 4.44)	1.87 (1.09, 3.19)	1.88 (1.10, 3.21)	1.90 (1.11, 3.27)
Q4 (2.196–2.737)	338	2.93 (1.79, 4.80)	1.58 (0.96, 2.85)	1.57 (0.87, 2.84)	1.59 (0.87, 2.90)
Q5 (≥2.741)	337	3.77 (2.15, 5.60)	1.77 (1.01, 3.11)	1.79 (1.01, 3.16)	1.79 (1.01, 3.19)
p for trend		<0.001	0.052	0.054	0.055

Note: OR, odds ratio; CI, confidence interval; MetS, metabolic syndrome.

^a^Model 1 was unadjusted for any covariate.

^b^Model 2 was adjusted for sex, age, education, income, smoking status, alcohol intake, exercise, aspartate aminotransferase level, alanine aminotransferase level, serum creatinine level, mercury level, and cadmium level.

^c^Model 3 was further adjusted for thyroid-stimulating hormone level.

^d^Model 4 was further adjusted for thyroid-stimulating hormone and creatinine-adjusted urinary iodine level.

## Discussion

In this study, we investigated the association between environmental exposure to lead and MetS considering the effect of TSH, which we hypothesized to be an intermediate factor that mediates the influence of lead on MetS. By analyzing a large representative sample of Korean adults who participated in the 2013 KNHANES, significant associations between blood lead, serum TSH levels, and MetS were found in the general Korean population. The associations were evaluated by multiple logistic regression models, and the results showed that increased levels of blood lead are positively associated with elevated levels of serum TSH. Moreover, the risk of MetS was higher in individuals with higher blood lead and serum TSH levels. After additional adjustment for serum TSH levels, the association between blood lead levels and MetS was evaluated, and the OR remained stable which suggests that serum TSH does not influence the effect of lead on MetS development.

The positive association between TSH levels and MetS has been established through numerous studies. It may in part be explained by the regulation of lipid metabolism by thyroid hormones. Overt hypothyroidism, which is characterized by increased TSH and decreased thyroxine (T4) levels, leads to an increase in serum cholesterol levels [24]. More specifically, thyroid hormones regulate the activity of some key enzymes in lipoprotein transport, which participate in the regulation of cholesterol contents. Therefore, altered levels of thyroid hormones can induce considerable changes in the synthesis of cholesterols [6]. This in turn affects serum lipid profiles, and the elevation of serum lipid concentrations lead to MetS. A study conducted in the Netherlands explored the association between thyroid dysfunction and MetS and identified a significant association between serum TSH levels and serum lipid levels even in the euthyroid range [24]. Another study based on a healthy Korean population showed significant positive correlations between serum TSH levels and the levels of total cholesterol, triglycerides, and low-density lipoprotein cholesterol. The results from this study showed that TSH concentrations were positively related to the prevalence of MetS with almost a doubled risk of MetS in people with higher TSH levels, and confirmed the influence of TSH in serum lipid profiles leading to the cause of MetS [4].

The results of this study also indicated that lead exposure is a risk factor of MetS. However, the mechanisms underlying the association between lead exposure and MetS is partially similar to that between TSH and MetS. Exposure to lead induces oxidative stress, which contributes to disturbances in systemic lipid metabolism [14, 15, 25, 26]. Oxidative stress due to lead exposure can induce alterations in serum lipid contents by stimulating lipid peroxidation or by enhancing the susceptibility of lipids to peroxidation [27]. Subsequently, elevated levels of serum lipid due to lead exposure increases the likelihood of MetS as shown in the effects of altered TSH levels on lipid profiles. Meanwhile, the increase in blood lead levels can induce an increase in serum TSH levels. This association was also confirmed by the findings of our study, which indicated the positive association between elevated blood lead concentrations and high serum TSH levels. These results correspond to those of previous studies, which reported the association between lead exposure and thyroid dysfunction. Although most of these studies were conducted in workers who are occupationally exposed to lead, they consistently revealed a positive association between blood lead and serum TSH levels [28, 29], and this positive relation was also observed with relatively low concentrations of blood lead [30]. Based on these results, we considered the possibility of a mediation effect by TSH and suggested that previous studies that reported the association between lead exposure and MetS could have been overestimating the effect of lead if TSH does have a mediation effect. However, after investigating the mediation effect of TSH on the relationship between lead and MetS, we have confirmed that TSH does not mediate the effect of lead exposure on MetS development. Hence, the effect of lead exposure on MetS can be considered as an independent effect, regardless of TSH levels.

The independent effect of lead exposure on MetS may be explained by the fact that the occurrence of MetS caused by lead exposure is rather complicated and involves some other factors, which do not completely overlap with those in the process of MetS development due to imbalance of TSH levels. Oxidative stress induced by lead exposure plays an important role in several biological processes in vivo. It may cause an increase in the production of proinflammatory mediators and lipid peroxidation, suppress nitric oxide (NO) levels, and alter calcium homeostasis, which in turn increase the likelihood of clinical disorders in the MetS cluster [14, 15]. Other factors influenced by lead exposure, other than that related to lipid profiles also have significant associations with MetS. Especially, suppression of NO is related to the development of MetS components such as insulin resistance, endothelial dysfunction, hypertriglyceridemia, and chronic adipose tissue inflammation [31]. NO participates in many important physiological processes, including the regulation of vasodilation and regional blood flow and mitochondrial biogenesis and function. Reduction in the bioavailability of NO can cause endothelial dysfunction, and impaired activity of isoenzymes that play a role in NO formation is closely associated with insulin resistance [31, 32]. In addition, calcium homeostasis is also correlated with the occurrence of MetS [33, 34]. Abnormal serum calcium levels may affect insulin sensitivity and insulin release, which in turn leads to an increase in the risk of diabetes and MetS [33]. Based on these explanations, there are several more pathways through which lead can cause MetS, besides the common pathway shared with TSH level modifications. While dysregulation of lipid metabolism is the overlapping pathway of MetS causation by lead and TSH, dysfunction of non-lipid factors which also link the association between lead and MetS are not yet proved to be related to TSH levels [35–40]. Therefore, the effect of lead on the occurrence of MetS can be regarded as an independent effect not mediated by TSHs.

The importance of this study is that we revealed the significant associations between lead exposure and TSH levels and that between lead exposure and MetS in a large representative sample of the general Korean population. Previous studies reporting the associations between two of the three variables mentioned above using nationwide data have mostly been conducted in the United States, and only a limited number of studies have been conducted in the general Asian population. Moreover, most studies on lead exposure in Korea have been conducted in industrial workers, and no study has reported the association between lead and TSH levels in the general Korean population. Hence, this was the first study to report the association between lead exposure and TSH levels in the general Korean population. Another remarkable strength of this study is that we noticed that TSH is closely related to both lead exposure and MetS and hypothesized the role of TSH as an intermediate factor. By examining the effect of TSH in the relationship between lead and MetS, we verified that TSH does not have an influence on the effect of lead exposure on MetS development and thereby eliminated the possibility that previous studies on lead and MetS could have overestimated the effect of lead exposure on MetS development.

There are some limitations to our study. First, as KNHANES data are cross-sectional survey data, the associations that we illustrated cannot represent a causal relationship. Second, we only utilized TSH as an indicator of thyroid function. KNHANES data also include the data on serum levels of fT4, which are also widely used as a measure of thyroid function, but we did not determine the effect of fT4 on the association between blood lead and MetS. However, TSH is independently affected by lead exposure regardless of other thyroid hormones [9, 29, 41], whereas the effect on other thyroid hormones is associated with the duration of exposure to lead [9]. Since KNHANES does not provide relevant data on the duration of exposure to environmental risk factors, it would be inappropriate to use fT4 as an indicator of thyroid function. Finally, blood lead mainly represents the degree of recent exposure as lead has a biological half-life of less than 30 days in the blood [27]. Therefore, blood levels of lead may not be a useful marker for determining the effect of long-term exposure to environmental lead on chronic diseases, such as MetS. Bone lead is a preferable biomarker of cumulative lead exposure instead of blood lead [42]. However, we used blood lead levels in our analysis because the data from KNHANES did not include data on bone lead levels. Yet, since the measurement error of exposure is likely to be non-differential, the actual association can be expected to be larger.

## Conclusion

A significant association between blood lead levels and MetS was observed in the general Korean population. Moreover, serum TSH levels were both positively associated with blood lead concentrations and MetS. The association between lead exposure and MetS was not attenuated after adjustment for TSH levels, suggesting that TSH does not mediate the effect of lead exposure on MetS development. These findings may indicate that lead has an independent effect on the pathogenesis of MetS. Further studies are needed to quantify the associations between lead and TSH in general populations and to examine the mechanism of MetS due to lead exposure.
